# Supplementary material for: Cryptosporidium infection of human small intestinal epithelial cells induces type III interferon and impairs infectivity of Rotavirus
Source: Gut Microbes. 2024 Jan 8;16(1):2297897. doi: 10.1080/19490976.2023.2297897 (PMC10793699; doi:10.1080/19490976.2023.2297897)
Supplement: Supplement.docx [file KGMI_A_2297897_SM9447.docx]

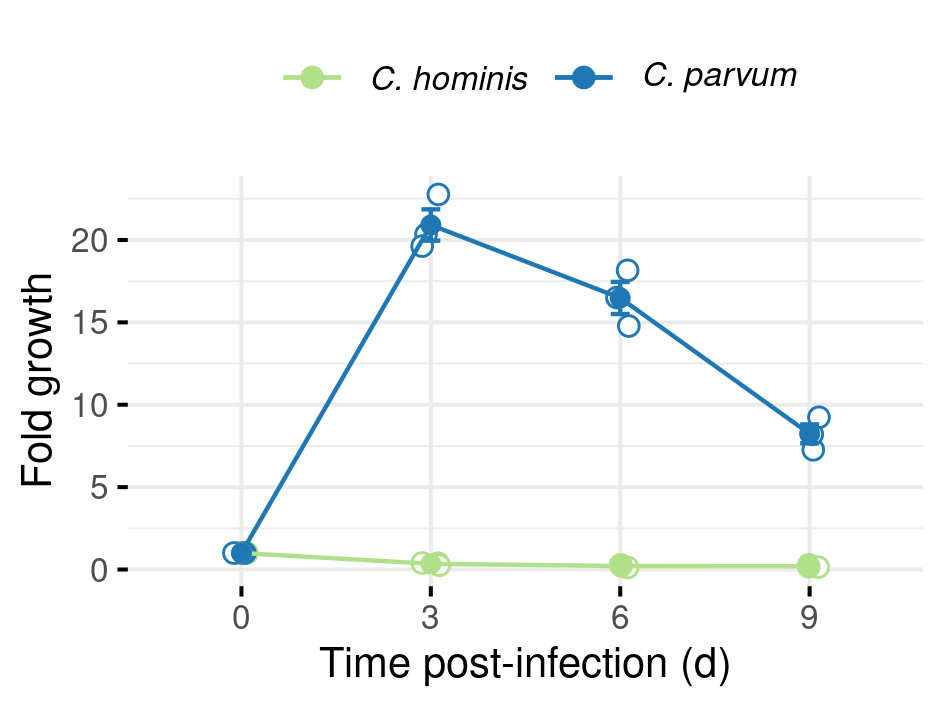


Fig. S1 – Growth of *C. parvum* and *C. hominis* in mouse ALI cultures over a 9-day period, as measured by qPCR. Mouse ALI does not sustain *C. hominis* growth. Mean ± S.E. N = 1 experiment, n = 3 technical replicates.


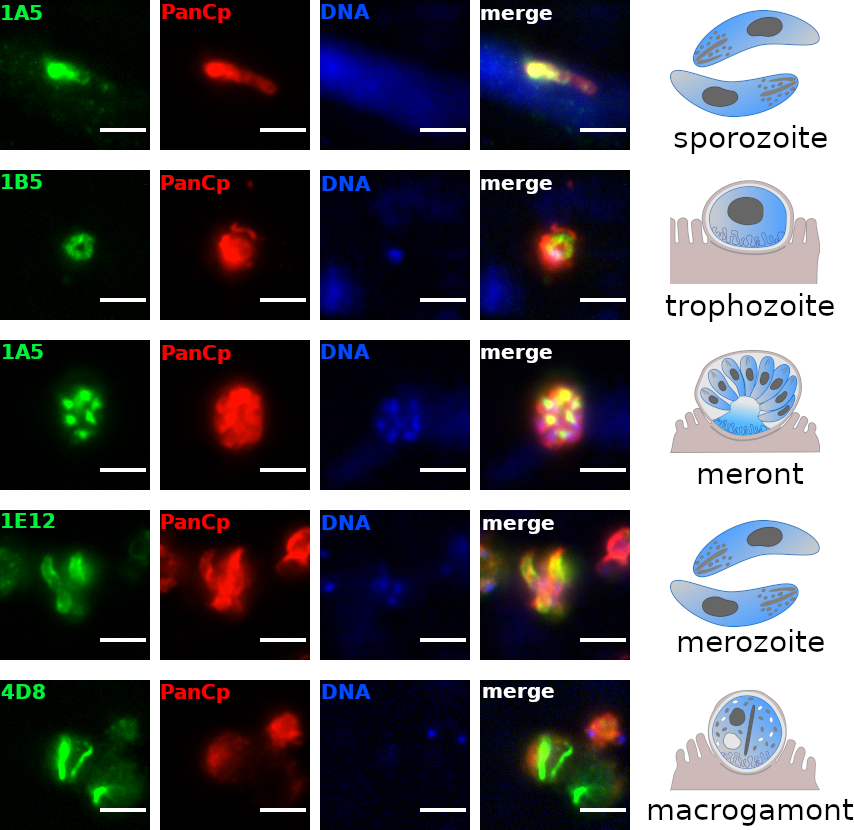


Fig. S2 – Immunostaining of various stages of *C. hominis* cultured on HCT-8 cells using a panel of previously described mAb to *C. parvum* (green) and a pan-*Cryptosporidium* polyclonal antibody (red). Scale bar = 3 μm.


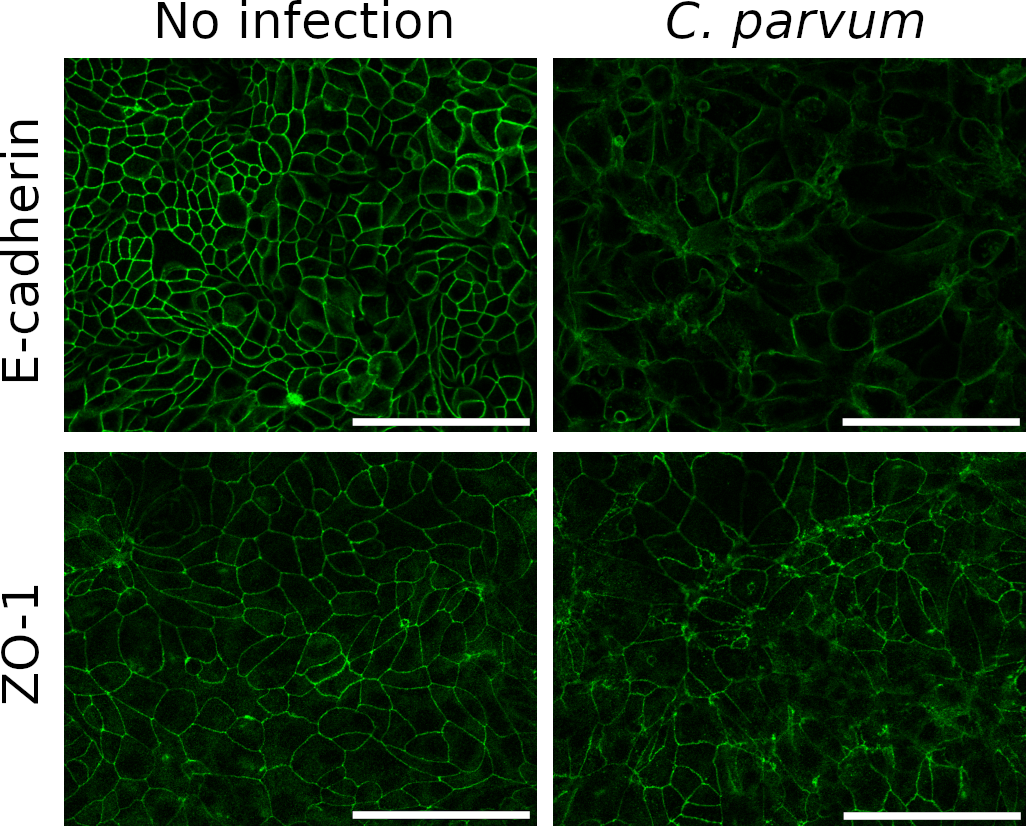


Fig. S3 – Immunostaining of HCT-8 transwell (E-cadherin) or coverslips (ZO-1) cultures showing tight junction (TJ; ZO-1, green) and adherens junction (AJ; E-cadherin, green) organization, with or without infection with *C. parvum* (not stained). Scale bar = 50 μm.


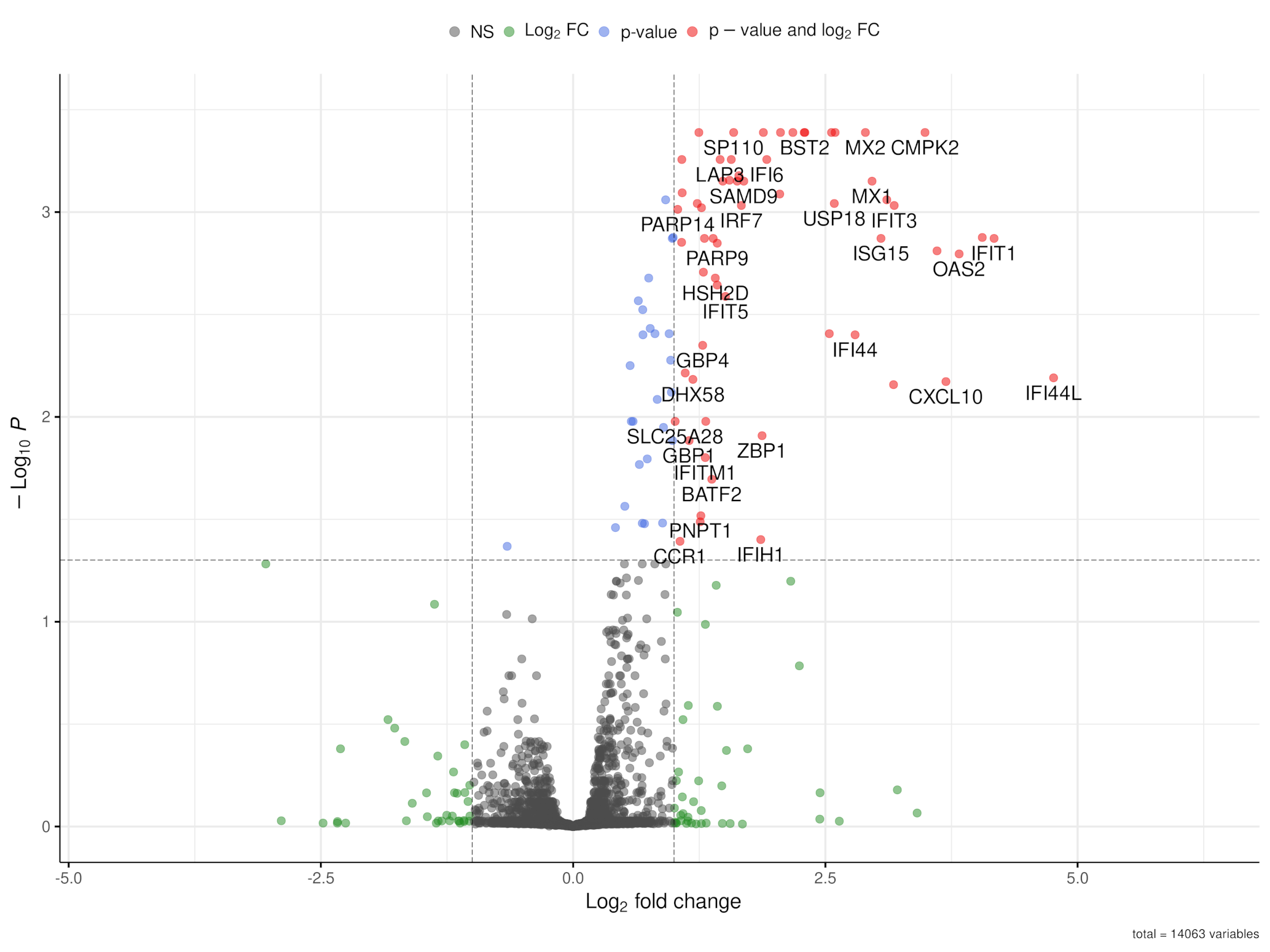


Fig. S4 – Volcano plot of differentially expressed genes in hALI 24 hr after infection with *C. hominis*. Genes up- or down-regulated by more than 2-fold and *P* < 0.05 are shown in red.
